# Structure of the Human Telomeric Stn1-Ten1 Capping Complex

**DOI:** 10.1371/journal.pone.0066756

**Published:** 2013-06-24

**Authors:** Christopher Bryan, Cory Rice, Michael Harkisheimer, David C. Schultz, Emmanuel Skordalakes

**Affiliations:** 1 Gene Expression and Regulation Program, The Wistar Institute, Philadelphia, Pennsylvania, United States of America; 2 Department of Chemistry, University of Pennsylvania, Philadelphia, Pennsylvania, United States of America; 3 Department of Biochemistry and Biophysics, University of Pennsylvania, Philadelphia, Pennsylvania, United States of America; Tulane University Health Sciences Center, United States of America

## Abstract

The identification of the human homologue of the yeast CST in 2009 posed a new challenge in our understanding of the mechanism of telomere capping in higher eukaryotes. The high-resolution structure of the human Stn1-Ten1 (hStn1-Ten1) complex presented here reveals that hStn1 consists of an OB domain and tandem C-terminal wHTH motifs, while hTen1 consists of a single OB fold. Contacts between the OB domains facilitate formation of a complex that is strikingly similar to the replication protein A (RPA) and *yeast* Stn1-Ten1 (Ten1) complexes. The hStn1-Ten1 complex exhibits non-specific single-stranded DNA activity that is primarily dependent on hStn1. Cells expressing hStn1 mutants defective for dimerization with hTen1 display elongated telomeres and telomere defects associated with telomere uncapping, suggesting that the telomeric function of hCST is hTen1 dependent. Taken together the data presented here show that the structure of the hStn1-Ten1 subcomplex is conserved across species. Cell based assays indicate that hTen1 is critical for the telomeric function of hCST, both in telomere protection and downregulation of telomerase function.

## Introduction

Telomeres are the guanosine rich DNA repeats at the ends of linear eukaryotic chromosomes that play a crucial role in protection and replication of the genome [1], [2]. An assortment of telomere-associated proteins allows the cell to address the special challenges of telomere maintenance and replication [3], [4], [5], [6]. In *S. cerevisiae*, the trimeric CST complex (*sc*CST, composed of Cdc13, *sc*Stn1 and *sc*Ten1) binds tightly and specifically to the telomeric G-overhang [7] and plays a critical role in telomere maintenance [7], [8], [9], [10]. G-strand binding by *sc*CST primarily depends on Cdc13, although telomeric DNA binding by *sc*Stn1 and *sc*Ten1 has also been proposed [11], [12], [13].

Cdc13 recruits telomerase to telomeres through binding to Est1, an interaction that is required for telomere extension *in vivo* [14], [15], [16], [17]. However, the *sc*CST complex negatively regulates telomere length by sequestering the telomeric overhang thus preventing access of telomerase to telomeres [9]. After G-strand extension, *sc*CST promotes telomeric C-strand fill-in by recruiting the DNA polymerase α-primase to telomeres; a process mediated by the N-terminal domain of Cdc13 and the C-terminal domain of Stn1 [10], [18]. In addition to its role in telomere replication, *sc*CST caps telomeres to prevent recombination and exonucleolytic degradation events that could lead to genomic instability and checkpoint-dependent cell-cycle arrest in the G2/M phase [7], [19], [20], [21], [22], [23]. Loss of any *sc*CST protein component leads to telomere phenotypes associated with uncapped telomeres [9], [10], [24].

Homologues of *sc*Stn1 and *sc*Ten1 were identified in fission yeast by Martin V. *et* *al.* [25] in 2007 and have been subsequently discovered in a wide range of species, including plants and vertebrates including humans [4], [26]. Like the *sc*CST, the hCST (Ctc1, hStn1 and hTen1) complex is predicted to contain multiple OB-folds and it binds telomeric overhangs with high affinity and some specificity [4], [27], [28], [29].

hStn1 and hCtc1 are also cofactors of DNA polymerase α-primase that promote C-strand synthesis at the telomeres [29,30,31. Loss of CST function in higher eukaryotes results in accumulation of excessive G-strand telomere DNA and the formation of extra-chromosomal t-circles, events associated with telomere uncapping [4], [27], [32]. Despite other similarities to the *sc*CST complex, there is currently no evidence to suggest that hCST recruits telomerase to telomeres. Instead, hCST blocks telomerase access to the G-overhang by inhibiting its interaction with TPP1 [28], a component of the shelterin telomere maintenance complex [6]. Loss of any component of hCST results in telomerase-mediated telomere lengthening [28]. In contrast to *sc*CST, some reports suggest a possible role for hCST in extra-telomeric DNA replication. hCST has been implicated in promoting genome-wide replication restart after fork stalling by promoting dormant replication origin firing [27], [33]. Overall, hCST plays a role in telomere replication, and may also have a more general role under conditions of replicative stress. Despite the shared similarities between *sc*CST and hCST, the low sequence identity and reported functional differences between the two complexes raise questions regarding their structural and functional conservation in telomere length regulation and chromosome end protection.

To further elucidate the role of hCST at the telomeres, we solved the structures of hTen1 in complex with the N-terminal domain of hStn1 (hStn1N), and the C-terminal domain of hStn1 (hStn1C) alone. These structures reveal that hStn1-Ten1 is a RPA-like complex and a structural homologue of *Schizosaccharomyces pombe* Stn1Ten1 (*sp*Stn1Ten1) and *Candida tropicalis* Stn1Ten1 (*ct*Stn1Ten1). DNA binding assays reveal that hStn1 shows a robust ssDNA binding activity while hTen1 binds ssDNA at a non-appreciable level (≥15 µM). Functional assays of hStn1 mutants defective for hTen1 binding show telomere signal-free ends, longer and fragile telomeres, phenotypes associated with telomere length deregulation and a dysfunctional *sc*CST complex. These data support a RPA-like DNA binding mechanism for hStn1-Ten1, and show that this complex plays a critical role in telomere maintenance.

## Results

### Structure of the hStn1-Ten1 Complex

**Figure 1:**
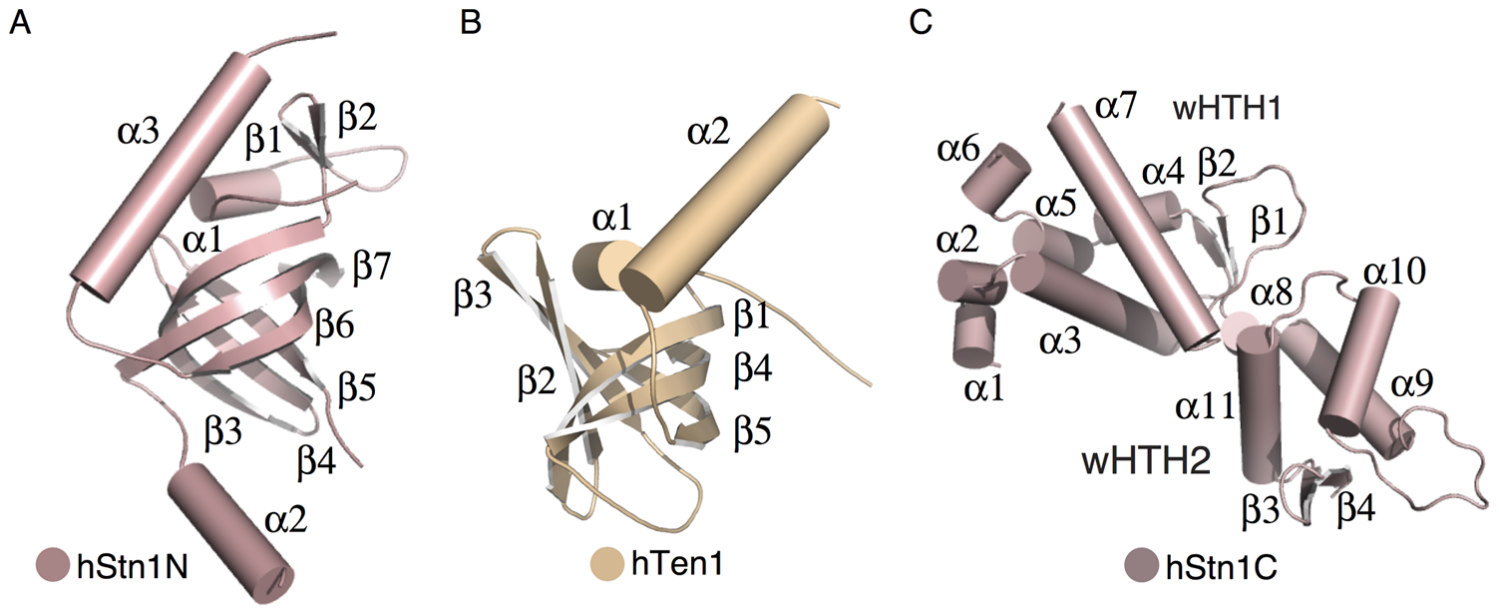
Crystal structures of hStn1 and hTen1. (**A**) The N-terminal domain of hStn1, (**B**) full-length hTen1, and (**C**) The C-terminal domain of hStn1.

To elucidate the function of the human telomeric Stn1-Ten1 complex in telomere biology, we prepared the full-length hStn1-Ten1 (**Figure S1A**), the truncated hStn1N-Ten1 (**Figure S1B**) and hStn1C (**Figure S1C**) proteins to homogeneity. We subsequently solved the structures of the hStn1N-Ten1 complex and hStn1C to 2.05 Å and 1.65 Å resolution respectively using mercury derivatives and the method of multi-wavelength anomalous dispersion (MAD) (**Table 1** **and** **2**). hStn1N adopts an OB fold, consisting of a β-barrel, composed of two three-stranded anti-parallel β-sheets (with β3 shared across both of the β-sheets), sandwiched by three α-helices (**Figure 1A**). An indentation on the surface of the protein forms the putative substrate-binding pocket (PBP) of the molecule. Helix α1 is located at the very N-terminus of the domain and caps one end of the β-barrel. Helix α2, composed of approximately twelve non-conserved residues, is located between strands β5 and β6 and is partially visible in the electron density. A striking feature of hStn1N is a long α-helix (α3) located at the opposite end of the substrate-binding pocket of the OB-fold and runs parallel to the plane of the β-barrel (**Figure 1A**). hTen1 adopts a similar OB fold consisting of five antiparallel β-strands folded into a β-barrel and flanked by two alpha helices. The first twelve N-terminal residues comprise a long coil that sits atop the surface of the β-barrel (**Figure 1B**) and plays an important role in hStn1-Ten1 assembly. Following the N-terminal coil is a short alpha helix (α1) positioned at the edge of the interface of the two β-sheets that form the β-barrel providing stability to the OB fold. The β-barrel contains the classic puckered surface that usually comprises the substrate-binding pocket of OB fold proteins. Located at the opposite end of the β-barrels’ puckered surface is a long C-terminal α-helix (α2) that spans the entire length of the β-barrel, like α3 of hStn1 (**Figure 1A and B**). The C-terminal domain of hStn1 consists of eleven α-helices and four β-strands organized into two distinct winged helix-turn-helix (wHTH) motifs (**Figure 1C**) similar to *sc*Stn1 [34], [35]. Surprisingly the RMSD between human and *sc*Stn1C (PDB ID: 3KEY and 3K10) is 6.1 Å [36]. However, the inflated RMSD observed between the two structures arises in large part from the overall organization of the two wHTH motifs and not the fold of the protein. Structural alignment of the hStn1C and *sc*Stn1C, wHTH1 motifs shows that the hStn1C wHTH2 motif is rotated about 25° away from the equivalent motif of *sc*Stn1C (**Figure S2B**) suggesting structural flexibility between the two motifs especially in their substrate-free state. However, structural comparison of the equivalent hStn1C and *sc*Stn1C (PDB ID: 3KEY or 3K10), wHTH motifs show RMSDs of 2.0 Å and 3.3 Å (DALI) for wHTH1 and wHTH2 respectively (**Figure S2C and S2D**), suggesting Stn1C structural conservation between the two organisms. The hStn1 wHTH2 is most similar to the wHTH of human RPA32 (PDB ID: 1DPU) with an RMSD of 2.0 Å (DALI) (**Figure S2E**). This result was unexpected given that the wHTH1 domain of *sc*Stn1 is most similar to the wHTH of RPA32 [**34**], [35].

**Table 1:** hStn1N-Ten1 Complex Data Collection and Refinement statistics.

Data collection	Native	Hg Derivative (MAD Phasing)	
		E1	E2
Wavelength (Å)	1.1	1.0076	1.1
Space group	P2_1_2_1_2	P2_1_2_1_2	P2_1_2_1_2
Cell dimensions			
*a*, *b*, *c* (Å)	130.6 58.1 87.6	129.8 55.9 89.0	130.0, 56.1 89.0
Resolution (Å)	20-2.05 (2.16-2.05)	50-2.15 (2.19-2.15)	50-2.15 (2.19-2.15)
*R*_sym_	7.5 (43.5)	6.3 (43.0)	6.6 (49.6)
*I*/*σ*/	9.7 (1.6)	19.5 (1.8)	17.7 (2.1)
Completeness (%)	100 (100)	99.9 (99.9)	99.8 (99.6)
Redundancy	5.8 (5.9)	3.4 (3.4)	3.4 (3.1)
**Phasing Analysis**			
Resolution (Å)			50-2.7
Mean figure of merit (FOM)			0.49
Score (solve)			28.8
Number of sites			8
**Refinement**			
Resolution (Å)	20-2.05		
No. reflections	40467		
*R*_work/_ *R*_free_	20.9/25.2		
No. atoms			
Protein	4215		
Water	190		
B-factors			
Protein	37		
Water	40		
R.m.s deviations			
Bond lengths (Å)	0.009		
Bond angles (°)	1.445		
Ramachandran plot (%) (Coot)			
Preferred	93.68		
Allowed	5.14		

**Table 2:** hStn1C data collection, phasing and refinement statistics.

Data Collection	Native	Hg Derivative (MAD Phasing)	
		E1	E2
Wavelength (Å)	1.1	1.0076	1.1
Space group	P2_1_2_1_2_1_	P2_1_2_1_2_1_	P2_1_2_1_2_1_
Cell dimensions			
*a*, *b*, *c* (Å)	28.8 76.6 114.4	28.8 76.5 113.9	28.8 76.7 114.1
Resolution (Å)	20-1.6 (1.69-1.6)	50-1.6 (1.69-1.6)	50-1.6 (1.69-1.6)
*R*_sym_	5.9 (48.6)	6.4 (50.2)	5.5 (29.9)
*I*/*σ*/	13.8(1.5)	13.6 (1.5)	14.9 (2.4)
Completeness (%)	99.9 (99.8)	96.4 (91.3)	95.1 (80.4)
Redundancy	5.6 (4.0)	3.0 (2.9)	2.9 (2.5)
**Phasing Analysis**			
Resolution (Å)			50-2.5
Mean figure of merit (FOM)			0.42
Score (solve)			30.5
Number of sites			2
**Refinement**			
Resolution (Å)	20-1.6		
No. reflections	32625		
*R*_work/_ *R*_free_	20.7/21.6		
No. atoms			
Protein	1350		
Water	98		
B-factors			
Protein	17.9		
Water	21.0		
R.m.s deviations			
Bond lengths (Å)	0.008		
Bond angles (°)	1.047		
Ramachandran plot (%) (Coot)			
Preferred	97.56		
Allowed	2.44		

**Figure 2:**
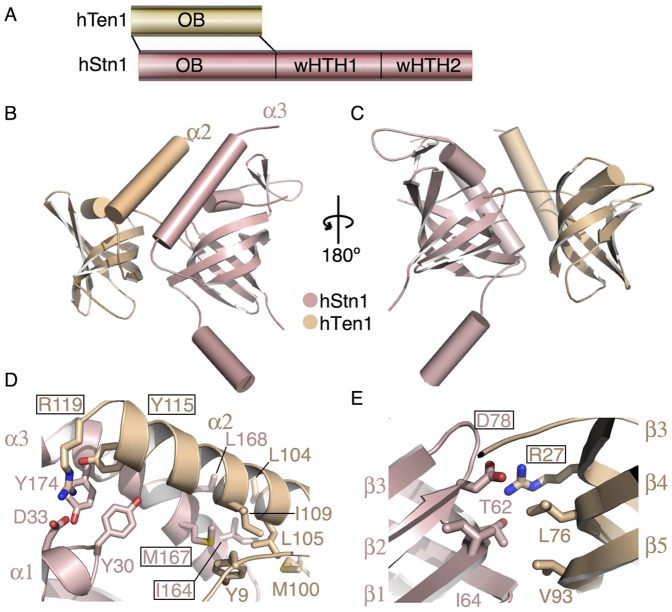
Structure of the hStn1N-Ten1 heterodimer. (**A**) Primary structures of hStn1 and hTen1 showing interacting domains. (**B**) Crystal structure of the hStn1-Ten1 dimer in cartoon representation looking down the interface of the two subunits. (**C**) View of the hStn1-Ten1 dimer rotated 180° to highlight the arrangement of OB fold putative DNA binding pockets. (**D**) Dimerization contacts between the hStn1N and full-length hTen1 C-terminal helices α3 and α2 respectively. Residues mutated in this study for ITC and cell based assays are shown in boxes. (**E**) Dimerization contacts between the β-barrels of hStn1N and hTen1.

Full-length hStn1 and hTen1 form a stable heterodimer via the N-terminal portion of hStn1 (**Figure 2A**). Contacts between the two proteins are mediated by extensive interactions between the C-terminal helices (α2 and α3 of hTen1 and hStn1 respectively) and β-barrels of the two proteins (**Figure 2B and C** **)**. These two helices are highly conserved among *sp*Stn1-Ten1, *ct*Stn1-Ten1 hStn1-Ten1 and RPA14-RPA32 where they are also involved in protein oligomerization [34], [37]. Many of the conserved residues making contacts between hStn1N and hTen1 are located on the C-terminal helices and the β-barrels of these proteins. In particular, residues V159, W160, I164, M167 and L168 of helix α3 and flanking coils of hStn1 form an extensive hydrophobic patch that interacts with the conserved residues, M100, L104, L105 and I109, of α2 and flanking coils of hTen1 (**Figure 2D**). Additional contacts between the α-helices of the two proteins are mediated by the conserved Y115 of α2 of hTen1. This residue is located at the interface of the two proteins and makes extensive hydrophobic interactions with the side chains of Y49, P171 and Y174 of hStn1N (**Figure 2D**). Additional contacts between the two proteins involve the surface of the β-barrels and the N-terminal tail of hTen1, which runs along the interface of the two domains and makes extensive interactions with both proteins (**Figure 2C**). In particular, R27 (β1) and R119 (α2) of hTen1 form salt bridges with D78 (β2) and D33 (α1) of hStn1, respectively (**Figure 2E**). The conserved residue M167 of hStn1 extends toward the interface between α2 and the β-barrel of hTen1 and makes extensive contacts with Y9 of the N-terminal coil and L105, A108 and I109 of α2 (**Figure 2D**). Interestingly, hStn1-Ten1 domain organization positions the substrate-binding pockets of each subunit on the same side of the heterodimer, creating an extensive substrate-binding pocket (**Figure 2C**).

A search in the PDB database using the Dali server shows that the structure of the hStn1N-Ten1 complex is strikingly similar to the *S. pombe* and *C. tropicalis* Stn1-Ten1 telomeric complexes with an RMSD of 2.1 Å and 2.9 Å respectively (**Figure S3A and B**), indicating significant structural conservation of these telomeric complexes between humans and *sp*Stn1Ten1 and *ct*Stn1Ten1. There is also a degree of structural conservation between hStn1N-Ten1 and the human RPA32-RPA14 complex (PDB ID:1QUQ) with an RMSD of 3.1 Å (**Figure S3C**).

### hStn1-Ten1 DNA Binding Properties

**Figure 3:**
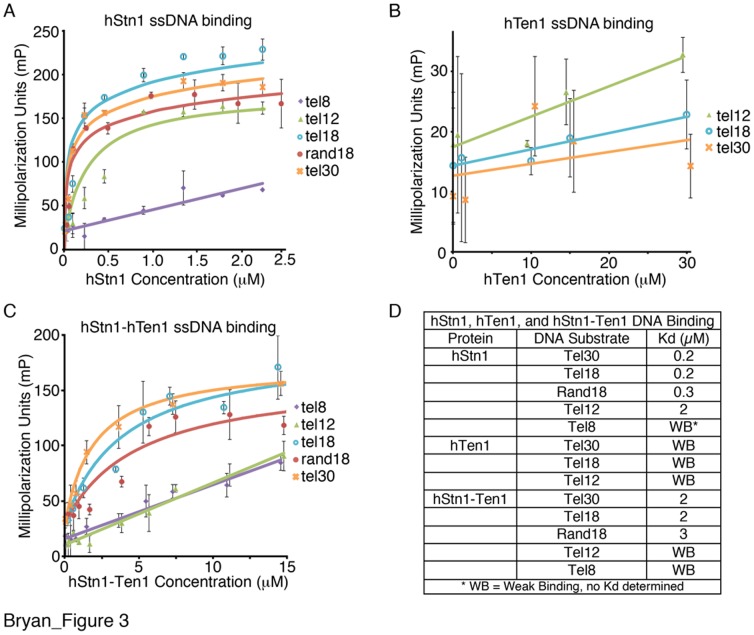
DNA binding properties of hStn1, hTen1 and the hStn1-Ten1 complex. Fluorescence Polarization (FP) data for (**A**) hStn1 (**B**) hTen1 and (**C**) the full length hStn1-Ten1 complex with ssDNA of various lengths (tel8, tel12, tel18, rand18, tel30– Table 3) (**D**) Table of hStn1, hTen1 and full-length hStn1-Ten1, ssDNA dissociation constants (K_d_) calculated from the FP data of panels A, B and C.

Given that hCST binds single stranded DNA and hStn1 and hTen1 adopt OB folds, we sought to determine whether one or both proteins contribute to the ssDNA binding activity of the hCST complex. Using fluorescence polarization assays, we tested whether full-length hStn1, hTen1, and the hStn1-Ten1 complex bound to telomeric and non-telomeric DNA oligomers of various lengths. hStn1 binds ssDNA substrates 18 bases or longer (telomeric (tel18) and non-telomeric (rand18) – **Table 3**) with an affinity of ∼0.2 µM (**Figure 3A**) while its affinity for probes shorter than 3 telomeric repeats was significantly decreased. For example it binds 2 telomeric repeats (tel12– **Table 3**) with ∼1 µM and its binding affinity for a 8mer (tel8– **Table 3**) was too weak to determine (**Figure 3D**).

**Table 3:** DNA oligos used in this study.

Oligo	Sequence
Tel30	TTAGGGTTAGGGTTAGGGTTAGGGTTAGGG
Tel18	TTAGGGTTAGGGTTAGGG
Rand18	GTTACGAAATACGGACAC
Tel12	TTAGGGTTAGGG
Tel8	TTAGGGTT

**Figure 4:**
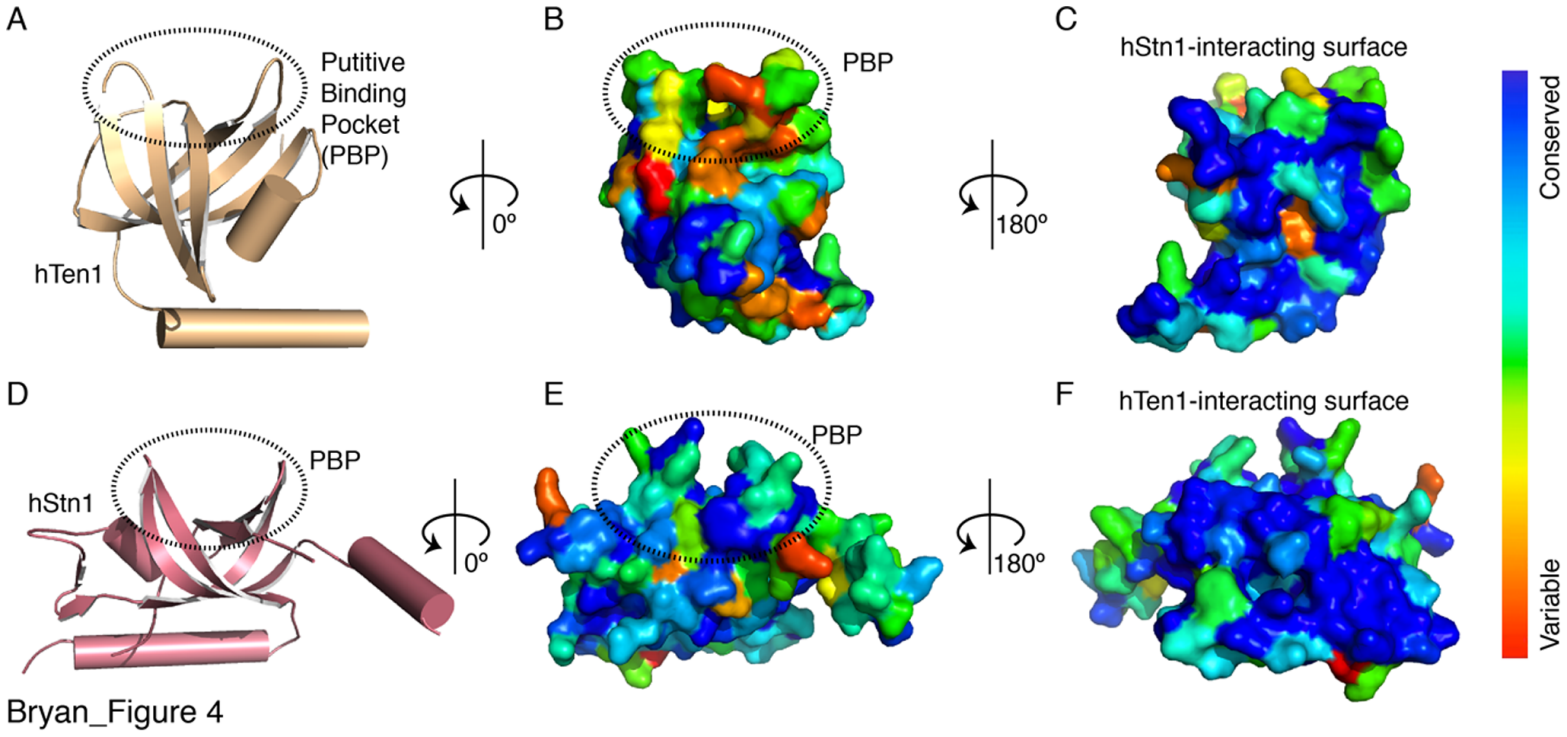
hStn1 and hTen1 surface aminoacid conservation. (**A**) Tertiary structure (cartoon) of hTen1 highlighting the putative DNA binding pocket (PBP) with a dashed circle. (**B** and **C)** Conservation map for the PBP and the hStn1-interacting surface of hTen1 respectively. Blue indicates residue conservation and red, residues that are variable. (**D**) Tertiary structure (cartoon) of hStn1 highlighting the PBP with a dashed circle. (**E** and **F**) Conservation map for the putative binding pocket of hStn1 and the hTen1-interacting surface respectively.

hTen1 also contains an OB fold with a similar structure to hStn1, but interacts with ssDNA much more weakly, as FP showed only a slight shift at protein concentrations of at least 15 µM or higher (**Figure 3B and D**). The ssDNA binding activity of hTen1 could not be detected using a tel30 probe, but was visible for a tel18 probe and best for a tel12 probe (**Figure 3B** **and** **Table 3**). This is likely due to the large dynamic range of small probes in FP experiments. The weak ssDNA binding activity of hTen1 is supported by structural conservation analysis, as the residues that form the putative DNA binding pocket (PBP) of hTen1 are poorly conserved (**Figure 4B** **)**. This suggests that hTen1 is not subject to evolutionary pressure to maintain a strong ssDNA binding activity. In fact, the only conserved surface area of hTen1 is involved in hStn1 binding (**Figure 4C**). In contrast, hStn1’s putative ssDNA binding pocket is composed of several highly conserved, solvent accessible residues (**Figure 4E**).

Interestingly, the hStn1-Ten1 complex exhibited non-specific ssDNA binding activity but with an affinity lower than that observed for hStn1 alone. For probes of at least 18 nucleotides or longer the binding affinity of hStn1-Ten1 is ∼2 µM, while we could not determine a K_d_ for the tel12 probe (**Figure 4C and D** **and** **Table 3**). The difference (10 fold) in DNA binding affinity for the tel18 between hStn1 alone and the hStn1-ten1 complex can be explained as follows; the minimum DNA length required for maximum hStn1-DNA binding (∼200 nM) is 18 bases, which is most likely sufficient to bind more than one hStn1 molecule. Binding of multiple hStn1 molecules to a single probe enhances the FP signal, leading to a lower calculated Kd. It is worth noting that the affinity of hStn1 for the tel12 probe (which most likely binds one hStn1 molecule) is comparable to the affinity of hStn1-Ten1 for tel18. It is therefore likely that a single hStn1 molecule has similar binding affinity to the hStn1-Ten1 complex.

### Mutants that Disrupt hStn1-Ten1 Dimerization in vitro

**Figure 5:**
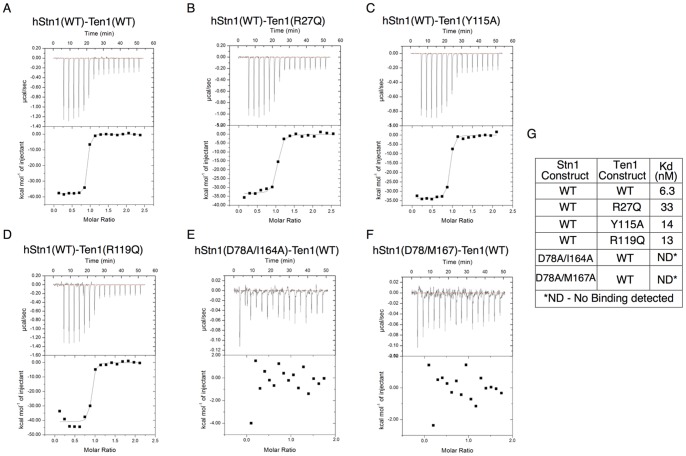
Isothermal titration calorimetry (ITC) data of hStn1 and hTen1 association. (**A**) hStn1(WT) with hTen1(WT). (**B**) hStn1(WT) with hTen1(R27Q). (**C**) hStn1(WT) with hTen1(Y115A). (**D**) hStn1(WT) with hTen1(R119Q). (**E**) hStn1(D78A/I164A) with hTen1(WT). (**F**) hStn1(D78A/M167A) with hTen1(WT). (**G**) Table of ITC values for the full length, WT, single and double mutant hStn1 and hTen1 proteins obtained from the curve fit of figures 6A–F.

To determine the role of hStn1-Ten1 complex assembly in telomere function we designed single and double mutants of conserved residues (**Figure S4A and B**) required for hStn1-Ten1 heterodimerization. We carried out isothermal titration calorimetry (ITC) experiments using pure, homogeneous, wild type and mutant proteins of hStn1 and Ten1 (**Figure S5**) in order to gauge the effect of these mutants on hStn1-Ten1 hetrodimerization. The wild type hStn1-Ten1 complex assembles with a dissociation constant of 6.3 nM (**Figure 5A**). Single mutants showed moderate loss (2–5.5 fold) of binding affinity while double mutants abolished hStn1N-Ten1 binding (**Figure 5B - F**). For example, the hTen1 (R27Q) mutation, which disrupts a salt bridge with D78 of hStn1, (**Figure 2E**) leads to a 5.5 fold loss (33 nM) of affinity between the two proteins (**Figure 5B and G**). The hTen1 (Y115A) mutation, which eliminates hydrophobic contacts with residues Y49, Y174 and P171 of hStn1 (**Figure 2D**), leads to a 2.5 fold (14 nM) loss of binding affinity (**Figure 5C**). The hTen1 (R119Q) mutation, which disrupts a salt bridge with D33 of hStn1 (**Figure 2D**), leads to a 2-fold reduction in binding affinity (13 nM) (**Figure 5D**). ITC experiments carried out using the double hStn1 mutants, (D78A/I164A) or (D78A/M167A) did not detect binding with wild-type hTen1 (**Figures 5E and F**). Both double hStn1 mutants disrupt the highly conserved salt bridge formed between D78 and R27 of hStn1 and hTen1 respectively (**Figure 2E**). I164A disrupts contacts with the hydrophobic patch of hTen1 consisting of residues M100, L104 and L105. M167A disrupts the extensive hydrophobic contacts involving Y9, L105, A108, and I109 of hTen1 (**Figures 2D**).

### Disruption of the hStn1-Ten1 Dimer Results in Elongated Telomeres

To investigate the role of hStn1-Ten1 assembly in telomere maintenance, we introduced the hStn1(D78A/M167A) and (D78A/M167A) double mutants that disrupt hStn1-Ten1 heterodimerization, into HEK 293T cells, and probed for telomere length defects using Southern blots. We knocked down endogenous hStn1 expression in HEK 293T cells by lentiviral infection with anti-hStn1 shRNA (shRNA-S2) (**Figure S6**). We prepared four hStn1 knockdown cell lines by co-infecting with a pLU vector containing: a) no hStn1 gene (hStn1-KD cell line), b) hStn1(WT) (hStn1-Rescue cell line) c) hStn1(D78A/I164A), and d) hStn1(D78A/M167A). We also created a mock-treated control cell line by co-infecting HEK 293T cells with a shRNA targeting green fluorescent protein (GFP), and the pLU vector that does not carry any gene. We also prepared a cell line expressing anti-hTen1 shRNA (shRNA-T1) and the pLU vector without any gene (hTen1-KD) in order to determine the effect of simple hTen1 knockdown (**Figure S6**). The ectopic hStn1 and hTen1 genes included silent mutations conferring resistance to the above shRNAs. None of the cell lines used in this study displayed any changes in growth rate or morphology after infection, and throughout the course of the experiment we applied antibiotic selection pressure to ensure that only successfully infected cells were able to propagate.

**Figure 6:**
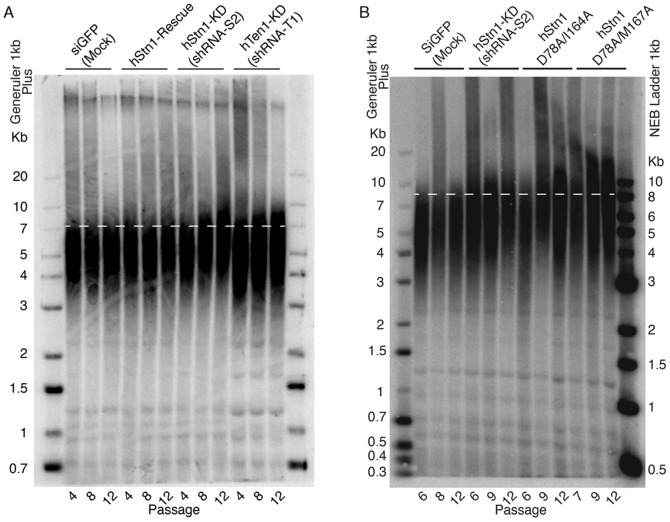
Southern blots analysis of telomeric DNA from cells carrying wild type (WT) or mutant hStn1 defective of hTen1 binding. (**A**) The gel shows telomere length at passages 4, 8 and 12 of cells carrying siGFP (Mock), hStn1-Rescue with WT protein, hStn1-KD (shRNA-S2) and hTen1-KD (shRNA-T1). The hStn1-KD (shRNA-S2) and double mutants defective of hTen1 binding show increased telomere length, compared to the siGFP (Mock). (**B**) The gel shows telomere length at passages 6, 7–9 and 12 of cells carrying siGFP (Mock), hStn1-KD (shRNA-S2) and the double mutants hStn1(D78A/I164A) and hStn1(D78A/M167A).

Southern blot analysis of DNA isolated from hTen1-KD, hStn1-KD of various passages (6, 9 and 12) showed a progressive increase in telomere length compared to mock-treated cells and hStn1-Rescue (**Figure 6A**), in agreement with previous reports [4], [28]. Surprisingly, the hStn1 double mutants hStn1(D78A/I164A) and (D78A/M167A) show slightly longer telomeres than the hStn1-KD (**Figure 6B**). The presence of longer telomeres in cells expressing hStn1(D78A/I164A) and (D78A/M167A), relative to knockdown alone, can be attributed to a dominant negative effect of the overexpressed hStn1 mutants. The hStn1 mutants were designed to specifically disrupt hTen1 but not Ctc1 binding. Saturation of hCtc1 with mutant hStn1 suppresses the activity of trace amounts of endogenous hStn1 produced under the shRNA knockdown, leading to a more severe phenotype.

### Disruption of the hStn1-Ten1 Complex Results in Chromosomal Abnormalities Associated with Dysfunctional Telomeres in vivo

Previous reports have shown that cells with defective hCST due to either hStn1 or hCtc1 knockdown show elevated levels of telomere signal-free ends [27] as well as fragile telomeres [33]. To further establish the role of the hStn1-Ten1 assembly in telomere maintenance, we asked if the hStn1, dimerization double mutants (D78A/I164A) and (D78A/M167A) exhibit a similar phenotype to simple knockdowns using fluorescence in situ hybridization (FISH) analysis. We prepared metaphase spreads of these cell lines by fixing the chromosomes to microscope slides with formaldehyde, and hybridizing telomeres with a Cy5-labelled peptide nucleic acid (PNA) probe targeting human telomeric repeats (PNA Bio, Inc.). Chromosomal DNA was stained with DAPI and photographs were taken using the 100× objective of a Nikon E600 upright fluorescent microscope.

**Figure 7:**
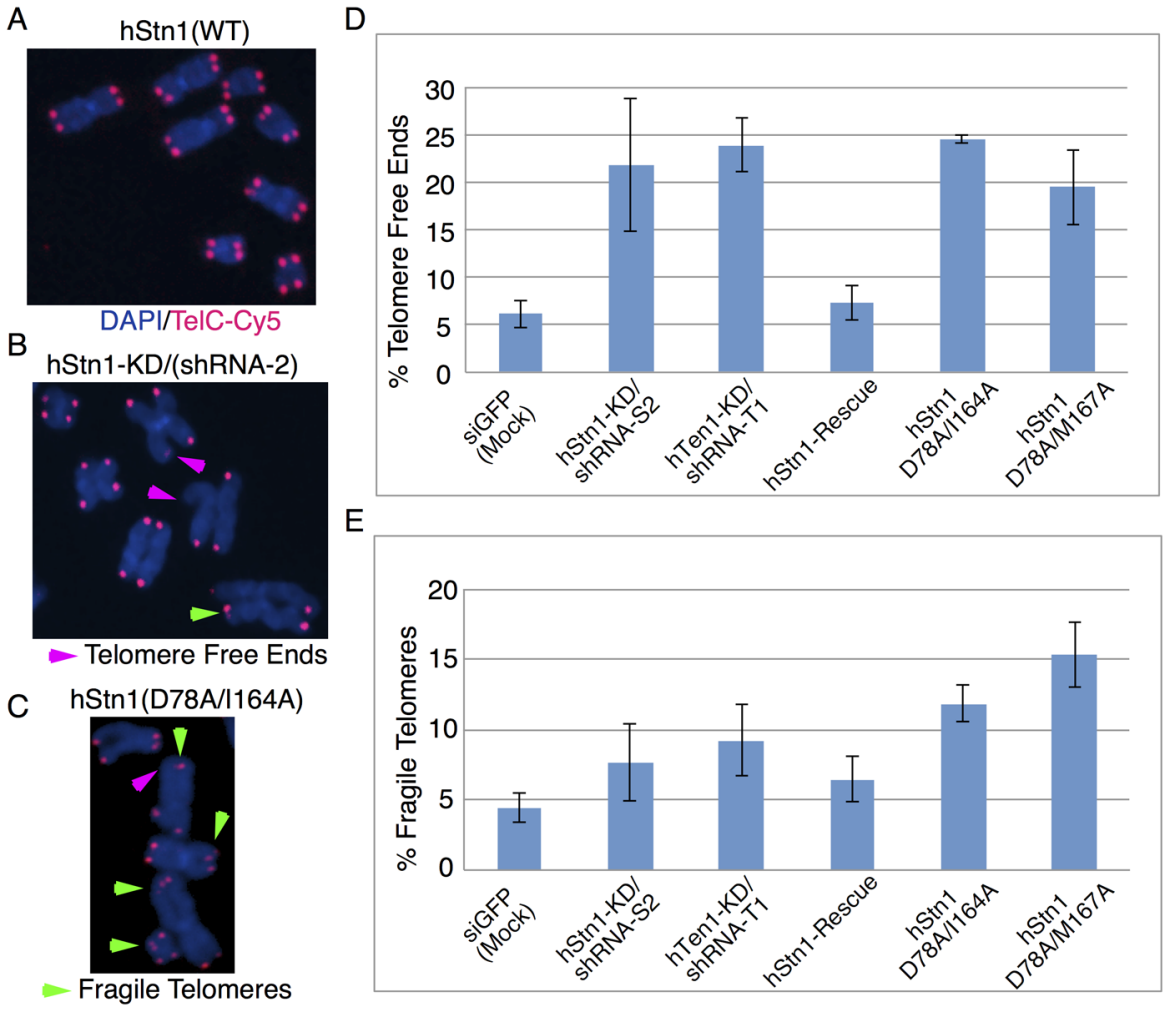
Fluorescence *in situ* hybridization (FISH) data of HEK 293 cells infected with: siGFP (Mock), hStn1-KD (shRNA-S2), hTen1-KD (shRNA-T1), hStn1-Rescue and hStn1 double mutants, hStn1(D78A/I164A) and hStn1(D78A/M167A),defective of hTen1 binding. (**A**) FISH of chromosomes in mock-treated (siGFP) cells display normal telomeres. (**B**) and (**C**) Telomere defects observed in hStn1 knockdown (hStn1-KD/shRNA-S2) and the double mutant hStn1(D78A/I164A) that disrupts hStn1-Ten1 association. Green arrows point to fragile telomeres, and pink to telomere free ends. (**D**) Bar graph showing the levels of telomere free ends observed in siGFP(mock), hStn1-Rescue and hStn1(D78A/I164A) or hStn1(D78a/M167A) double mutants defective of hTen1 binding. (**E**) Bar graph showing the levels of fragile telomeres observed in siGFP(mock), hStn1-Rescue and hStn1(D78A/I164A) or hStn1(D78a/M167A) double mutants and mutant hStn1 defective of hTen1 binding. Error bars show the standard deviation from 3 independent experiments. An average of ∼1000 chromosomes were counted in each experiment.

We counted the frequency of telomere signal-free ends, chromosome fusions, and fragile telomeres three weeks (passage 8) after infection. Knockdown of hStn1 or hTen1 led to elevated levels of chromosomes with missing telomeres (**Figure 7A, B and C**). This phenotype was rescued by reintroducing hStn1(WT), but not the hStn1 mutants (D78A/I164A and D78A/M167A) defective of hTen1 binding. Mock-treated (wild type) cells showed 6.2% of chromosomes with missing telomeres, compared with 21.9% in hStn1-KD, and 24.0% in hTen1 knockdown (**Figure 7D**). Reintroduction of hStn1(WT) to hStn1-KD cells reduced the number of missing chromosomes to 7.4%, while overexpression of the hStn1(D78A/I164A) or (D78A/M167A) double mutants led to a significant increase in the number of missing telomeres (24.6% and 19.5% respectively) (**Figure 7D**). We also found elevated levels of fragile telomeres in cells overexpressing hStn1 mutants defective for hTen1 dimerization (**Figure 7A, B and C** **)**. However, the levels of fragile telomeres in hStn1 and hTen1 knockdown cells were not significantly elevated over mock treated cells or hStn1-Rescue (**Figure 7E**). The increased number of TFE chromosomes observed for the hStn1 dimerization mutants (D78A/I164A) and (D78A/M167A) compared to hStn1 and hTen1 knockdown cell lines can most likely be explained by a dominant negative phenotype of the overexpressed mutant as discussed earlier. We did not observe a significant change in the rate of telomere fusions across any of our cell lines.

## Discussion

The identification of a human homologue of yeast CST in 2009 posed an interesting challenge for the telomere field as we need to rethink existing telomere capping models in vertebrates [4]. The results presented here provide evidence supporting structural and functional conservation of the CST complex across distant species, and provide insight into the role of the hStn1-Ten1 complex at telomeres. Structural overlays of hStn1-Ten1 with *sp*Stn1-Ten1 (PDB ID 3KF6) and *ct*Stn1-Ten1 (PDB ID 3KF8) show a surprisingly high degree of similarity between these complexes, given the evolutionary distance and low sequence identity between these species. We observed that the hStn1-Ten1 complex has striking structural similarities to both *sp*Stn1-Ten1 and *ct*Stn1-Ten1. This close structural relationship indicates that they likely carry out a conserved function at telomeres.

The fact that Stn1-Ten1 and RPA are structurally similar and share some functional aspects of DNA processing [30], [38], [39], [40] raises the question as to why cells need both complexes. Current evidence suggests that CST acts as a telomere specific version of the RPA complex, promoting efficient replication of telomeres [11]. hCST binds preferentially to the mammalian telomeric G-strand [28], and has been shown to localize to ∼20% of telomeres [4]. Telomeric DNA poses a challenge to the normal replication machinery due to its repetitive sequence and the formation of G-quadrupex structures upon unwinding duplex DNA [41], [42], [43]. It may be the case that RPA is unable to resolve these structures and assist in the polα - dependent DNA replication, so a specialized protein is required to promote efficient telomere replication. In support of this idea, disruption of CST has been shown to lead to delayed telomere replication and formation of telomere doublets, which may indicate replication fork stalling and collapse in the telomeric region [27]. CST may have an additional function in protecting telomere ends from DNA damage response by excluding RPA from binding to telomeric DNA, as RPA is capable of activating ATR-mediated DNA damage response, an activity that is undesirable at telomeres [44]. This notion is supported by the fact that in *S. cerevisiae* and humans CST association with telomeres reaches a maximum in the late S to early G2/M phase when G-overhangs are longest [28], [45], [46].

Structural data reveal subtle yet important differences between hStn1-Ten1 and Rpa32-Rpa14 that may provide an explanation to the above ideas. The most glaring difference between hStn1 and RPA32 is that hStn1 has 2 wHTH motifs C-terminal to the OB fold, while RPA32 only has one. wHTH2 of hStn1 is structurally similar to the wHTH of RPA32, so the presence of wHTH1 may partially account for differential function between RPA and CST. In both RPA and Stn1, the OB fold is a ssDNA-binding domain while the C-terminal domain is important for protein-protein interactions. The additional wHTH motif in hStn1 may allow interaction with a different set of proteins that function at telomeres such as Ctc1. Functional differences between hCST and RPA could also be attributed to lack of sequence identity and size of the RPA70 (616 aminoacids) and hCtc1 (1217 aminoacids), the large subunits of each complex respectively. There is little mechanistic and no structural data on hCtc1, so further studies are necessary to determine the role that it is playing in the function of the complex.

The data presented here shows that cell lines defective of hStn1-Ten1 heterodimerization exhibit telomere dysfunction in the form of elongated telomeres (**Figure 6**), telomere signal-free ends, and fragile telomeres (**Figure 7**), phenotypes associated with defective hCST [4], [27]. This reinforces functional homology between hCST and *sc*CST because both complexes are critical for telomere length regulation [9], [25], [47]. Our FP assays show that hStn1 binds ssDNA with a Kd of 200 nM alone and 2 µM when in complex with hTen1. This data establishes a role for the hStn1 protein in ssDNA binding, which facilitates telomere capping by hCST. In contrast, hTen1 alone showed only a slight shift at protein concentrations of ≥15 µM (**Figure 3B**). Although hTen1 alone binds ssDNA extremely weakly, its localization to the telomeres by the Ctc1-Stn1 complex would enhance hTen1 - DNA binding by positioning the protein in proximity to the telomeric overhang. The structure of the hStn1-Ten1 complex supports this notion as the organization of the hStn1-Ten1 OB folds aligns the PBPs so ssDNA substrate could easily interact with both proteins (**Figure 2C**). However the weak DNA binding affinity of hTen1 for ssDNA and the lack of amino acid conservation at its PBP raises important questions regarding the precise role of this protein in telomere maintenance. One possibility is that hTen1 is indeed involved in ssDNA binding, which would suggest a conserved ssDNA binding mechanism with the *sc*Stn1Ten1 complex [11]. *sc*Stn1 has been reported to bind ssDNA with a 2 µM affinity, compared to >6 µM for *sc*Ten1 [12], [13]. hCST-bound telomeric DNA must be accessible for C-strand synthesis by pol-α, which requires hCST to hand-off ssDNA. The function of the homologous RPA complex has been proposed to depend on sequential binding and release of ssDNA by multiple OB domains [48]. The strongest-binding OB domains of RPA are at the 5′ end of a bound substrate and the weakest at the 3′ end, with RPA32 (hStn1 homolog) on the far 3′ end [49]. Release of weakly associated ssDNA from hTen1 may be the first step in sequential unbinding of the OB folds in the hCST complex to allow hand-off of a ssDNA substrate.

Another possibility is that the weak ssDNA binding activity of hTen1 observed here is not biologically relevant and hTen1 has a different role in hCST dependent telomere maintenance. The recently published structure of the 30 nucleotide binding mode of the RPA complex (RCSB ID: 4GOP) [49] provides a possible model for cooperative DNA binding between subunits of the hCST complex. The OB fold of RPA32 (hStn1 homologue) is a DNA binding domain that is involved in the 30 nucleotide binding mode of RPA, and helps the complex to achieve its 0.1 nM binding affinity for ssDNA while RPA14 (hTen1 homologue) is not involved in high-affinity DNA binding [49]. It's possible then that hTen1 carries out primarily a structural role in hCST assembly thus enhancing Ctc1 and hStn1 [4] DNA binding via promoting the proper assembly of the trimeric CST complex. Without effective ssDNA binding hCST is not in position to cap telomeres effectively, leading to telomere elongation (**Figure 6**) and telomere damage (**Figure 7**) phenotypes observed in cells defective for hStn1-Ten1 complex formation. hTen1 may also act as a steric block, which prevents hStn1 and possibly Ctc1 from interacting with potential binding partners such as Polα. This notion is further supported by the lack of conserved solvent-exposed residues on hTen1 except for those involved in hStn1 binding (**Figure 4B and C**), Perhaps in the absence of hTen1, hStn1 and Ctc1 can interact with polα to promote C-strand synthesis.

## Materials and Methods

### Protein Expression and Purification

We designed the hStn1N construct using sequence alignment and secondary structure prediction with the PHYRE server [50]. The hStn1N gene consisting of residues 18–184, and carrying the his-MBP (Maltose Binding Protein) fusion tag at the N-terminus was overexpressed in Rosetta (DE3) pLysS cells (Millipore) using 1 mM isopropyl-β-D-thiogalactopyranoside (IPTG; Gold Biotechnology) for 5 hours at 20°C. hTen1 with a N-terminal his tag was overexpressed in BL21-CodonPlus(DE3) RIPL cells (Stratagene) using 1 mM IPTG for 3 hours at 20°C. Cells were harvested by centrifugation and mixed together prior to lysis by sonication in 0.5 M KCl, 25 mM Tris, 15 mM imidazole, 5% glycerol, 0.1 mM phenylmethylsulfonyl fluoride (PMSF), 0.1 mM benzamidine, pH 7.5 (buffer A). The hStn1-Ten1 purified using Superflow Ni-Nitrilotriacetic acid (Ni.NTA; Qiagen) column, followed by an amylose resin (NEB) column. The fusion tags were cleaved by TEV overnight at 4°C. Tandem Poros-HS and HQ columns (Perspective Biosystems) were used to remove residual contaminants and a Superdex S75 (GE Healthcare) to remove any aggregates. The full-length hStn1 was overexpressed and purified using the same procedure as that used for the hStn1N-Ten1 complex.

### Protein Crystallization and Data Collection

#### hStn1N-Ten1 crystallization

We concentrated the purified hStn1N-Ten1 complex to 18 mg/mL and dialyzed it for 3 hours in a buffer containing 100 mM KCl, 5 mM Tris, 1 mM TCEP, pH 7.5. hStn1N-Ten1 crystals of the orthorhombic space group P2_1_2_1_2 grew under the sitting drop vapor diffusion method at room temperature in one week from 1 µL drops containing the protein complex and 0.8 M AmSO_4_, 0.1 M citric acid pH 4.0 and 5% jeffamine M-600. Streak seeding into drops prepared by mixing 1 µL of hStn1N-Ten1 with 1 µL of 0.6 M AmSO4, 0.2 M Citric Acid pH 4.0, 5% jeffamine M-600, optimally reproduced the crystals. Crystals were harvested using a cryoprotectant consisting of 0.6 M AmSO_4_, 0.2 M Citric Acid pH 4.0, 5% jeffamine M-600, 30% ethylene glycol. Data were collected at the National Synchrotron Light Source (NSLS) X25 beamline and processed using HKL2000 (**Table 1** **and** **2**). The crystals contained two hStn1-Ten1 dimers in the asymmetric unit.

#### hStn1C crystallization

We concentrated the purified hStn1C to 41 mg/mL and dialyzed it for 3 hours in a buffer containing 100 mM KCl, 5 mM Tris-HCl, 1 mM TCEP, pH 7.5. hStn1C crystals of the P2_1_2_1_2_1_ space group grew under the microbatch method at room temperature in two weeks from 1 µL drops containing hStn1C and 1.6 M ammonium phosphate dibasic, 100 mM Tris pH 8.5 and 3% ethylene glycol. Crystals were harvested using a cryoprotectant consisting of 1.6 M ammonium phosphate dibasic, 100 mM Tris-HCl, pH 8.5, 3% ethylene glycol and 30% glycerol. Data were collected at the National Synchrotron Light Source (NSLS) X25 beamline and processed using HKL2000 (**Table 1** **and** **2**). The crystals contained one hStn1C molecule in the asymmetric unit.

### Structure Determination and Refinement

We used the method of multi-wavelength anomalous dispersion (MAD) experiment and mercury derivatized crystals to obtain initial phases. Mercury derivatives of hStn1N-Ten1 and hStn1C were prepared by incubating the crystals with 5 mM of MeHgCl for 15 minutes. Mercury sites for hStn1N-Ten1 and hStn1C were found using SOLVE [51] (**Table 1** **and** **2**), and refined in MLPHARE [52] with phase extension to 2.05 Å and 1.6 Å resolution using a native dataset. Density modification and model building was done in RESOLVE [53], [54] with two-fold non-crystallographic symmetry (NCS) for hStn1-Ten1. The final model was built in COOT [55] and refined using REFMAC [56] (**Table 1** **and** **2**). The atomic coordinates and structure factors have been deposited in the Protein Data Bank (RCSB - www.pdb.org) under the accession numbers 4JOI and 4JQF.

### Isothermal Titration Calorimetry (ITC)

We carried out ITC experiments on a MicroCal iTC200 (GE). Purified full-length hStn1 and hTen1 (WT and mutants) were buffer exchanged into (300 mM KCl, 25 mM Tris-HCl, pH 7.5, 1 mM DTT, 5% glycerol). hTen1 was injected at a concentration of 125 µM into the cell containing hStn1 at 12 µM. For the ITC runs, the cell of the calorimeter was kept at 20°C, and the volume of each injection was 2.47 µL. Analysis of ITC data used the Origin analysis software (GE Healthcare) to obtain binding constants and ratios.

### Fluorescence Polarization (FP)

15 binding reactions were prepared with 1 mg/mL BSA 100 mM NaCl, 5 mM MgCl_2_, 1 mM DTT, 5% glycerol, 2.5 nM 6-FAM labeled DNA oligo, and various concentrations of purified full length hStn1, hTen1, or hStn1-Ten1 complex. Reactions were mixed at room temperature in triplicate, transferred to an optiplate 384, and fluorescence polarization signal was measured using an Envision Xcite Multilabel Plate Reader (Perkin Elmer).

### Human Cell Culture

Human cell culture studies were carried out in HEK 293T cells. The hStn1, hTen1 genes in the pLU vector and shRNA in the pLKO.1 vector were delivered using lentiviral infection of the HEK 293T cells. We prepared lentiviral particles by lipofectomine 2000® (Invitrogen) transfection of HEK 293T cells with the pLU or pLKO.1 and lentiviral production vectors. pLKO.1 vectors carrying the hStn1 shRNAs and puromycin resistance were obtained from the Sigma Mission shRNA library (shRNA-S1, shRNA-S2, shRNA-S3, shRNA-S4 and shRNA-S5 correspond to TRCN 127870, 128123, 128703, 128801, and 129006 respectively). Four hTen1 shRNAs were also tested from the Mission shRNA library (shRNA-T1, shRNA-T2, shRNA-T3, shRNA-T4 correspond to TRCN 337345, 447411, 337412, 371156) (**Figure S6**). The ectopic WT and mutant hStn1 and hTen1 genes used in this study were designed to carry silent mutations conferring resistance to the selected shRNAs. For protein expression we used pLU-EF1A-iBlast (pLU) vector, carrying blasticidin S resistance. Growth media was spiked with 5 µg/mL blasticidin S, and 2 µg/mL puromycin.

### Western Blot

We tested the effectiveness of the Sigma Mission shRNAs against hStn1 and hTen1 in western blots using FLAG or hStn1 and hTen1 specific antibodies as well as RT-PCR. All three assays showed the same result. For the western blots, 3×10^6^ HEK 293T cells were lysed in 300 µL of pre-chilled RIPA buffer (50 mM Tris-HCl pH 7.5, 150 mM NaCl, 0.1% SDS, 0.5% Sodium Deoxycholate, 1% Triton X-100). Lysates were centrifuged for 15 minutes at 20,000×g and the supernatant saved. Protein supernatants were quantified using a Bradford assay [57], and 20 µg of each sample was run on a polyacrylamide gel. Protein blotted onto a polyvinylidene fluoride (PVDF) membrane (Perkin Elmer Health Sciences) by electrophoretic blot at 200 mA for 3 hours at 4°C in 20% methanol, 125 mM Tris-HCl, 1.25 M glycine and 0.5% SDS. The membrane was blocked with TBST (100 mM Tris-HCl, pH 7.5, 2 M NaCl, 0.5% Tween-20) and 5% bovine serum albumin (BSA), washed TBST, and then incubated overnight with monoclonal anti-FLAG antibody M2 produced in mouse (Sigma) in TBST at 4°C. The membrane was further washed with TBST, incubated with the horseradish peroxidase (HRP)-conjugated anti-mouse IgG antibody (GE Healthcare) for 2 hours, and washed again with TBST. The chemiluminescent HRP reaction was activated using SuperSignal West Pico Substrate (Thermo Scientific), and the image developed with CL-exposure film (Thermo Scientific).

### Southern Blot Analysis of Telomere Length

We extracted the genomic DNA from HEK 293T cells using the Qiagen QIAamp® DNA Mini Kit. We digested 10 µg of genomic DNA with 1.5 µl (15 U) of AluI (Invitrogen) and 3 µl (15 U) of MboI (NEB) endonucleases for 24 hours at 37°C. Samples were ethanol precipitated overnight at −20°C and pelleted by centrifugation, washed with 75% ethanol, and resuspended in 30 µl of DNase free water. We loaded 3 µg of each sample onto a 0.7% 1X Tris Acetate EDTA (TAE) agarose gel and run in 1× TAE buffer for 6 hours at 120 V. The gel was then washed with 0.25 M HCl, denaturing solution consisting of 0.5 M NaOH, 1.5 M NaCl, followed by neutralization buffer 0.5 M Tris-HCl, 3 M NaCl, pH 7.5. After washing, the DNA was transferred from the gel to a hybridization transfer membrane (Genescreen Plus™, Perkin Elmer Health Sciences) using capillary blotting. DNA was then UV cross-linked to the membrane using a UV Stratalinker 1800 (Stratagene), and hybridized with 0.2 nM of ^32^P labeled single stranded DNA probe (TTAGGG)_4_ overnight in 15 mL church buffer (7% SDS, 0.25 M Na_2_PO_4_ pH 7.2, 1 mM EDTA, 1% w/v BSA). The non-hybridized probe was removed by washing with buffer containing 20 mM Na_2_PO_4_ pH 7.2, 1% w/v SDS and 1 mM EDTA and the membrane was exposed to a phosphorimager overnight for sample imaging.

### Fluorescence in situ Hybridization (FISH)

HEK 293T cells were grown to 70% confluence on a 10 cm plate and treated with 100 µg/ml of colcemid for 4 hrs. Cells were then trypsinized to detach them from the plate, pelleted and treated in a hypertonic environment (75 mM KCl for 30 minutes at 37°C) to rupture them. The cells were fixed in 10 ml of 3∶1 methanol:acetic acid solution and stored at 4°C. Cells were dropped on frosted microscope slides (Thermo Scientific), rehydrated in coplin jars filled with phosphate buffered saline (PBS), fixed in 4% formaldehyde (Sigma), permeabilized with 1 mg/ml pepsin in 10 mM glycine, pH 2.0 at 37°C (Sigma), and fixed again with 4% formaldehyde. Slides were then dehydrated in 70%, 95%, then 100% ethanol successively, air dried, and hybridized with 20 µL of 200 nM telomeric-Cy5 peptide nucleic acid (PNA) probe (TelC-Cy5 - PNA biosciences) in 70% formamide, 10 mM Tris pH 7.5, 0.5% Odyessy blocking buffer (LiCor) according to the manufacturer’s instructions. Slides were stained with DAPI and imaged using a Nikon E600 upright microscope.

## Supporting Information

Figure S1 **Expression and purification data of the hStn1-Ten1 complex.** Size exclusion chromatogram and SDS PAGE analysis of (**A**) the full-length hStn1-Ten1 complex. (**B**) the hStn1N-Ten1 complex (**C**) the C-terminal domain of hStn1 (hStn1C).
(TIF)

Figure S2 **Structural homologs of hStn1C.** (**A**) Overall structure of the C-terminal domain of hStn1 with secondary structure elements labeled. (**B**) Structural alignment of hStn1C (pink) with *sc*Stn1C (green – PDB ID: 3KEY and 3K10). (**C**) and (**D**) Independent structural alignments of the two winged helix turn helix motifs of hStn1 (wHTH1 and wHTH2) and *sc*Stn1C. (**E**) Alignment of the wHTH2 motif of hStn1C (pink) with that of RPA32 (blue - PDB ID: 1DPU).
(TIF)

Figure S3 **Structural homologs of the hStn1-Ten1 complex.** Structural alignment of hStn1N-Ten1 (wheat cartoon) with: (**A**) *sp*Stn1N-Ten1 (green - PDB ID: 3KF6) (**B**) *ct*Stn1-Ten1 (red - PDB ID: 3KF8) and (**C**) RPA32-RPA14 (blue - PDB ID:1QUQ).
(TIF)

Figure S4 **Stn1, Ten1 and RPA sequence alignments.** (**A**) hStn1 sequence alignment with other Stn1 and RPA32; residues mutated in this study are shown in red color. (**B**) hTen1 sequence alignment with other Ten1 and RPA14; residues mutated in this study are shown in green color.
(TIF)

Figure S5 **SDS-PAGE gel analysis of purified hStn1 and hTen1 wild type and mutant proteins used in ITC experiments.** Lanes: 1) Markers 2) hStn1(WT) 3) hStn1(D78A/I164A) 4) hStn1(D78A/M167A) 5) hTen1(WT) 6) hTen1(R27Q) 7) hTen1(Y115A) 8) hTen1(R119Q).
(TIF)

Figure S6 **Western blots analysis of hStn1 and hTen1 expression in HEK 293T cellines.** (**A**) Anti-flag tag Western blot showing the effect of five hStn1 shRNAs on ectopic hStn1 overexpression in the HEK 293T cells. Lanes: 1) Markers 2) hStn1 (WT) 3) shRNA-S1 4) shRNA-S2 5) shRNA-S3 6) shRNA-S4 and 7) shRNA-S5. (**B**) Anti-flag Western blot testing shRNA resistance of 3 different hStn1 genes carrying silent mutations designed to prevent binding of shRNA-S1, 2 and 4. Lane 1) Markers; Lanes 2–7, HEK 293T cells co-infected with 2) shRNA-S1 resistant hStn1 3) siGFP and shRNA-S1 resistant hStn1 4) shRNA-S2 resistant hStn1 5) siGFP and shRNA-S2 resistant hStn1 6) shRNA-S4 resistant hStn1 7) siGFP and shRNA-S4 resistant hStn1 (**C**) Anti-flag tag Western blot showing the effect of anti-hTen1 shRNAs on ectopic hTen1 overexpression in the HEK 293T cells. Lanes: 1) Markers 2) untransfected HEK 293T cells 3) hTen1-pLU 4) shRNA-T1 5) shRNA-T2 6) shRNA-T3 and 7) shRNA-T4.
(TIF)
