# Supplementary figures and images for: Structure of the Human Telomeric Stn1-Ten1 Capping Complex
Source: PLoS One. 2013 Jun 24;8(6):e66756. doi: 10.1371/journal.pone.0066756 (PMC3691326; doi:10.1371/journal.pone.0066756)

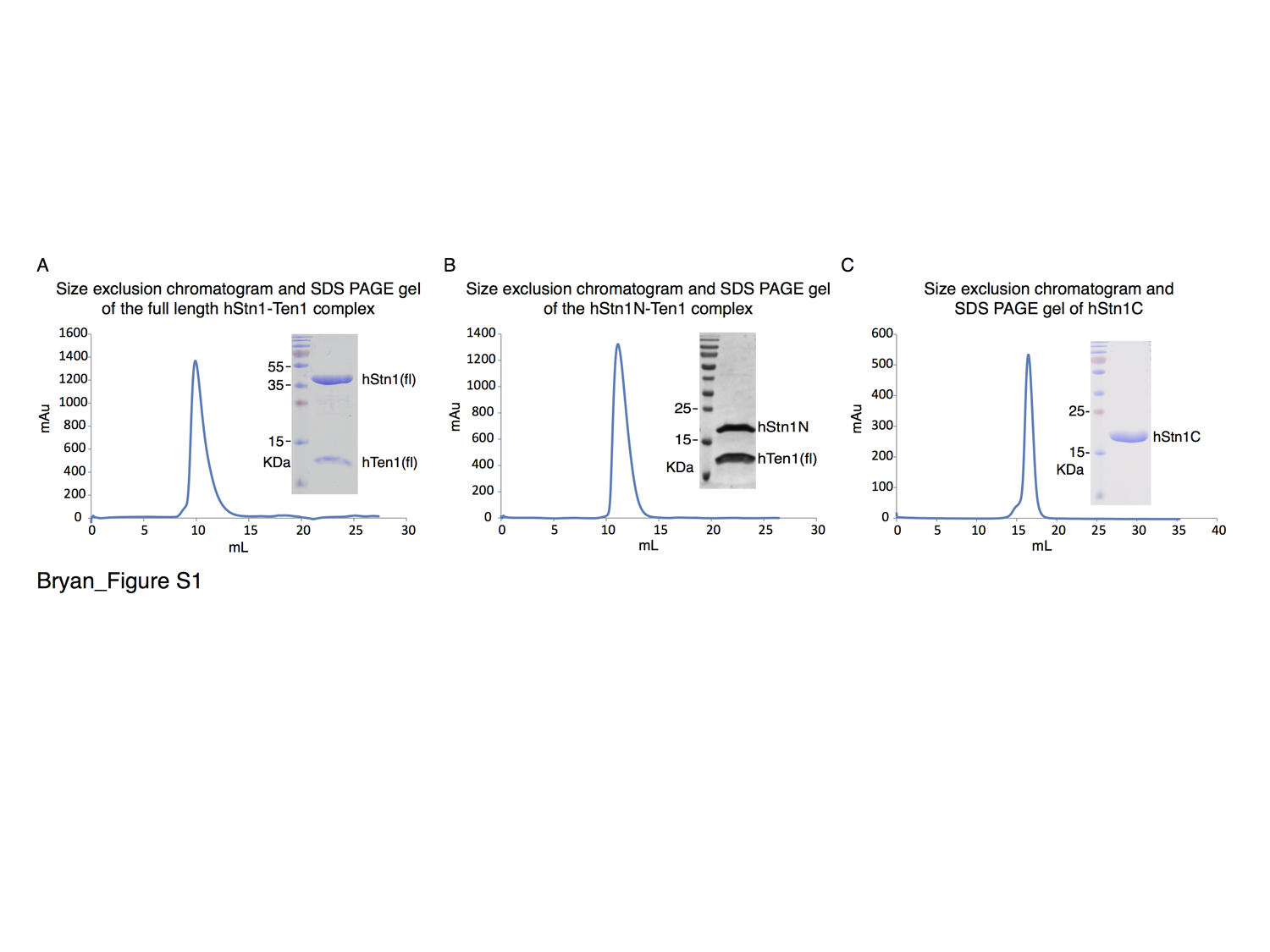

Supplement: Figure S1 — Expression and purification data of the hStn1-Ten1 complex. Size exclusion chromatogram and SDS PAGE analysis of (A) the full-length hStn1-Ten1 complex. (B) the hStn1N-Ten1 complex (C) the C-terminal domain of hStn1 (hStn1C). (TIF) [file pone.0066756.s001.tif]

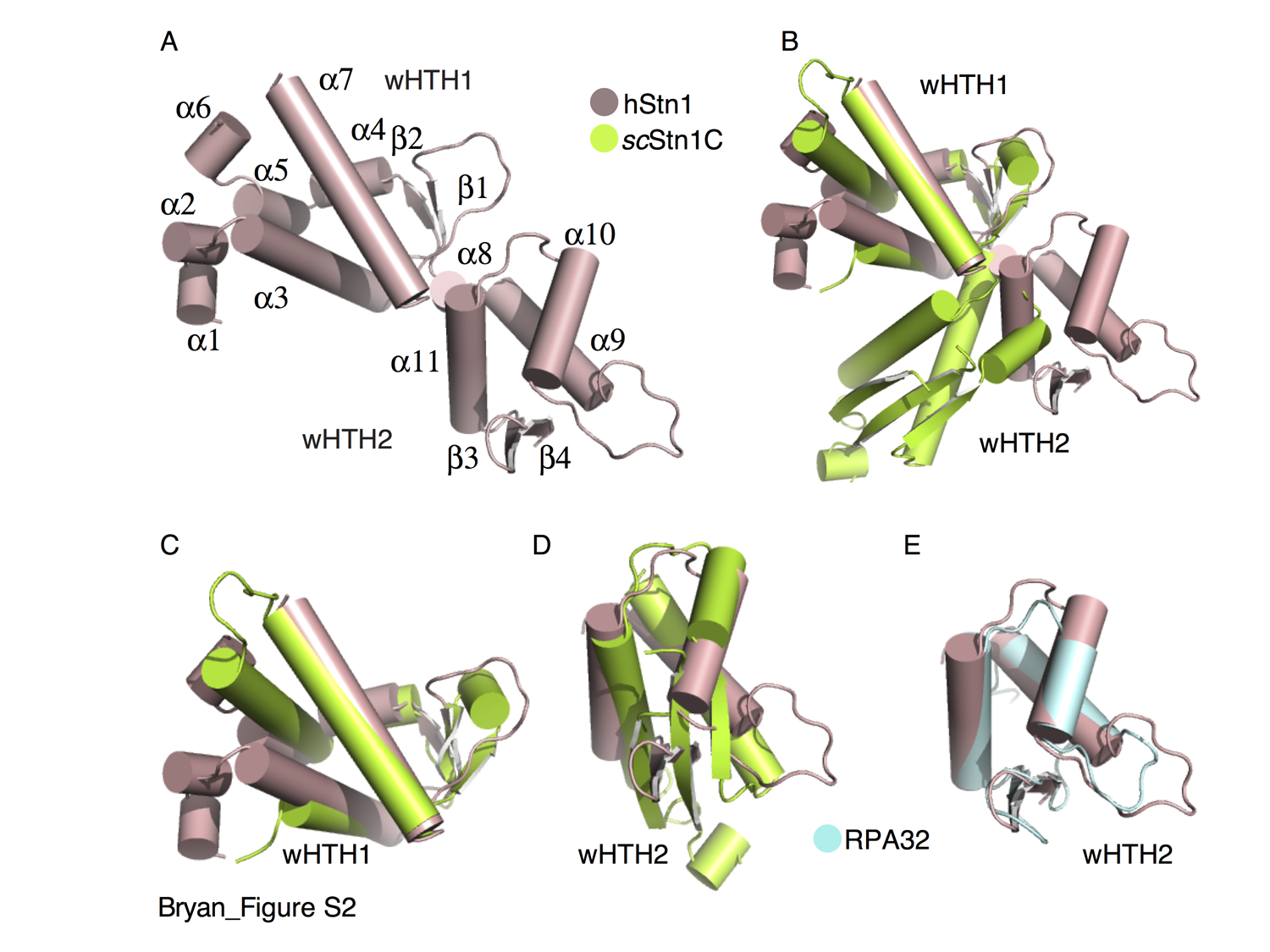

Supplement: Figure S2 — Structural homologs of hStn1C. (A) Overall structure of the C-terminal domain of hStn1 with secondary structure elements labeled. (B) Structural alignment of hStn1C (pink) with scStn1C (green – PDB ID: 3KEY and 3K10). (C) and (D) Independent structural alignments of the two winged helix turn helix motifs of hStn1 (wHTH1 and wHTH2) and scStn1C. (E) Alignment of the wHTH2 motif of hStn1C (pink) with that of RPA32 (blue - PDB ID: 1DPU). (TIF) [file pone.0066756.s002.tif]

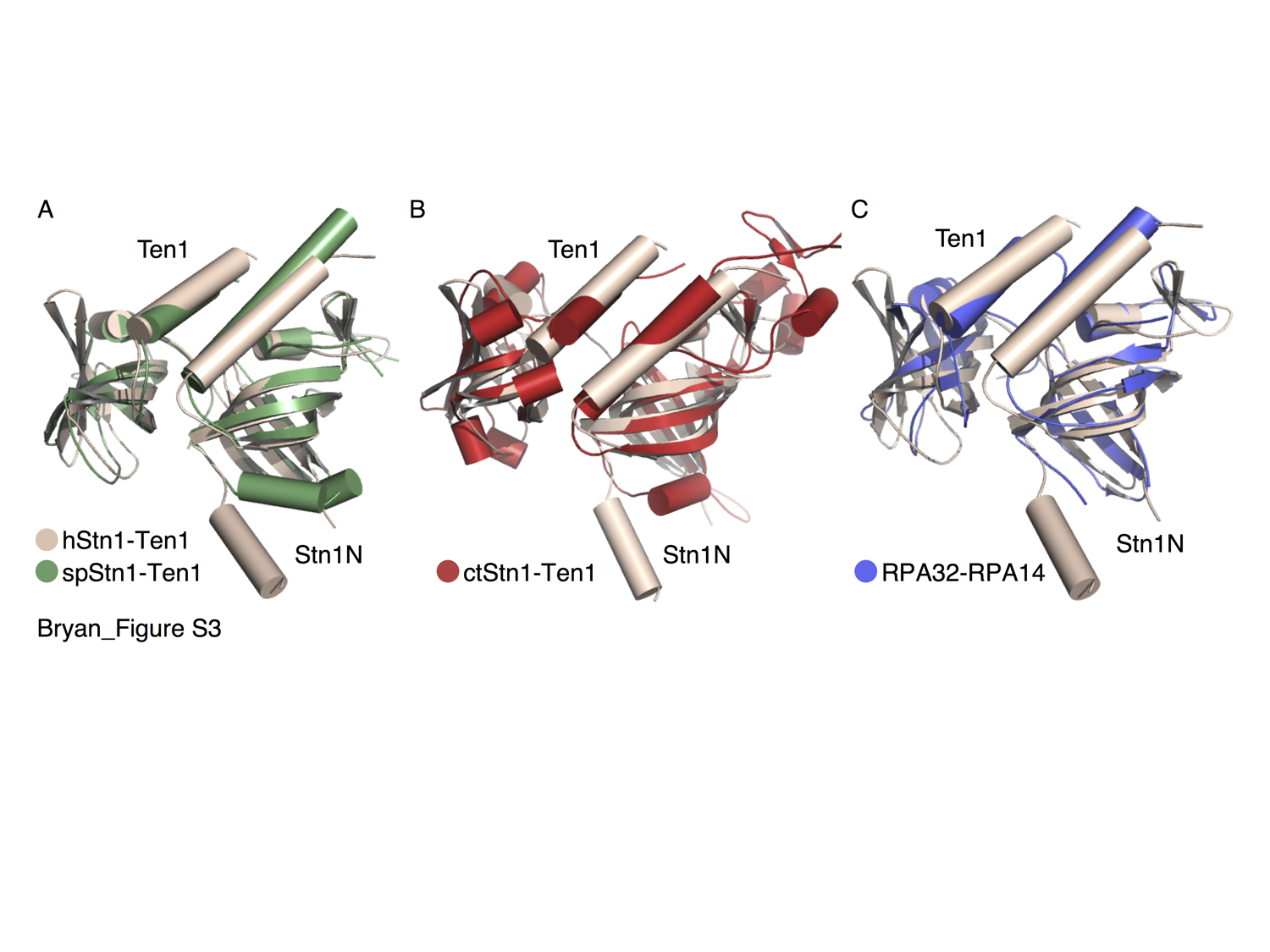

Supplement: Figure S3 — Structural homologs of the hStn1-Ten1 complex. Structural alignment of hStn1N-Ten1 (wheat cartoon) with: (A) spStn1N-Ten1 (green - PDB ID: 3KF6) (B) ctStn1-Ten1 (red - PDB ID: 3KF8) and (C) RPA32-RPA14 (blue - PDB ID:1QUQ). (TIF) [file pone.0066756.s003.tif]

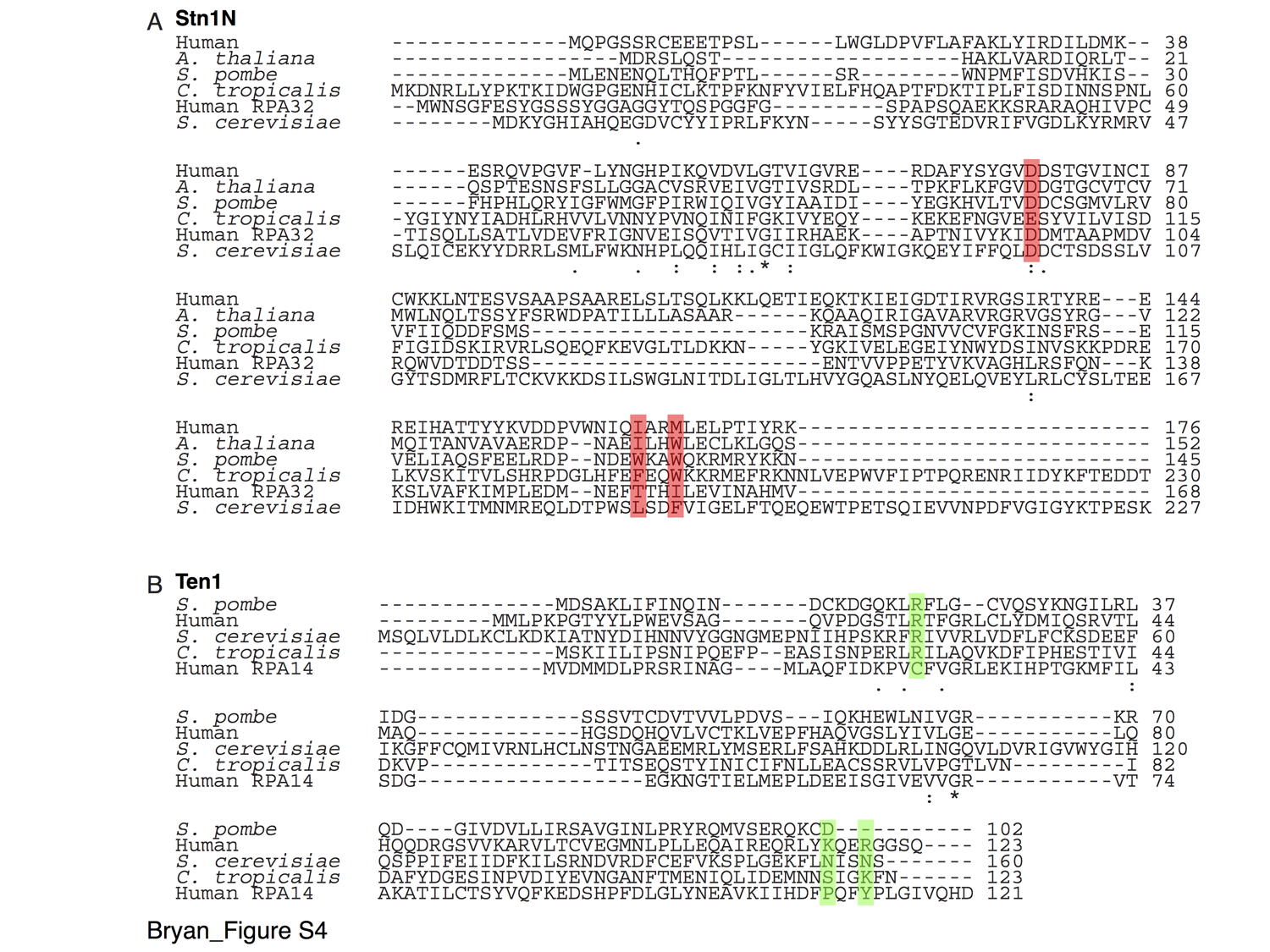

Supplement: Figure S4 — Stn1, Ten1 and RPA sequence alignments. (A) hStn1 sequence alignment with other Stn1 and RPA32; residues mutated in this study are shown in red color. (B) hTen1 sequence alignment with other Ten1 and RPA14; residues mutated in this study are shown in green color. (TIF) [file pone.0066756.s004.tif]

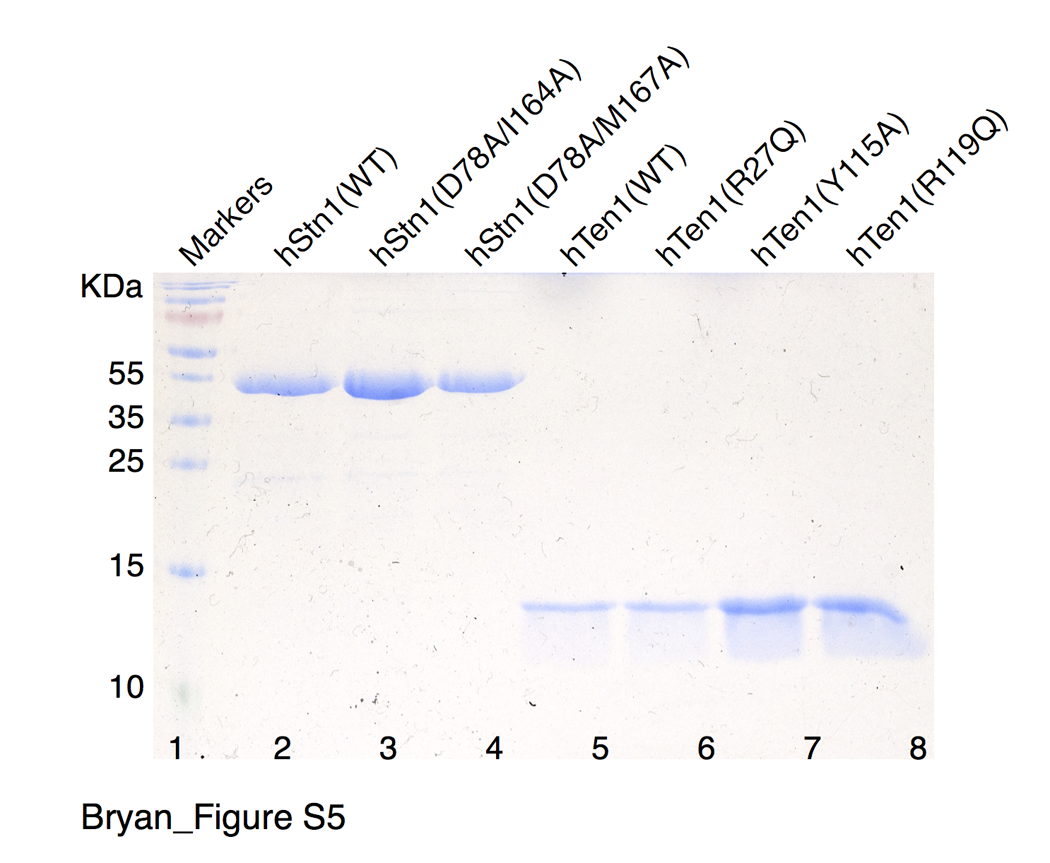

Supplement: Figure S5 — SDS-PAGE gel analysis of purified hStn1 and hTen1 wild type and mutant proteins used in ITC experiments. Lanes: 1) Markers 2) hStn1(WT) 3) hStn1(D78A/I164A) 4) hStn1(D78A/M167A) 5) hTen1(WT) 6) hTen1(R27Q) 7) hTen1(Y115A) 8) hTen1(R119Q). (TIF) [file pone.0066756.s005.tif]

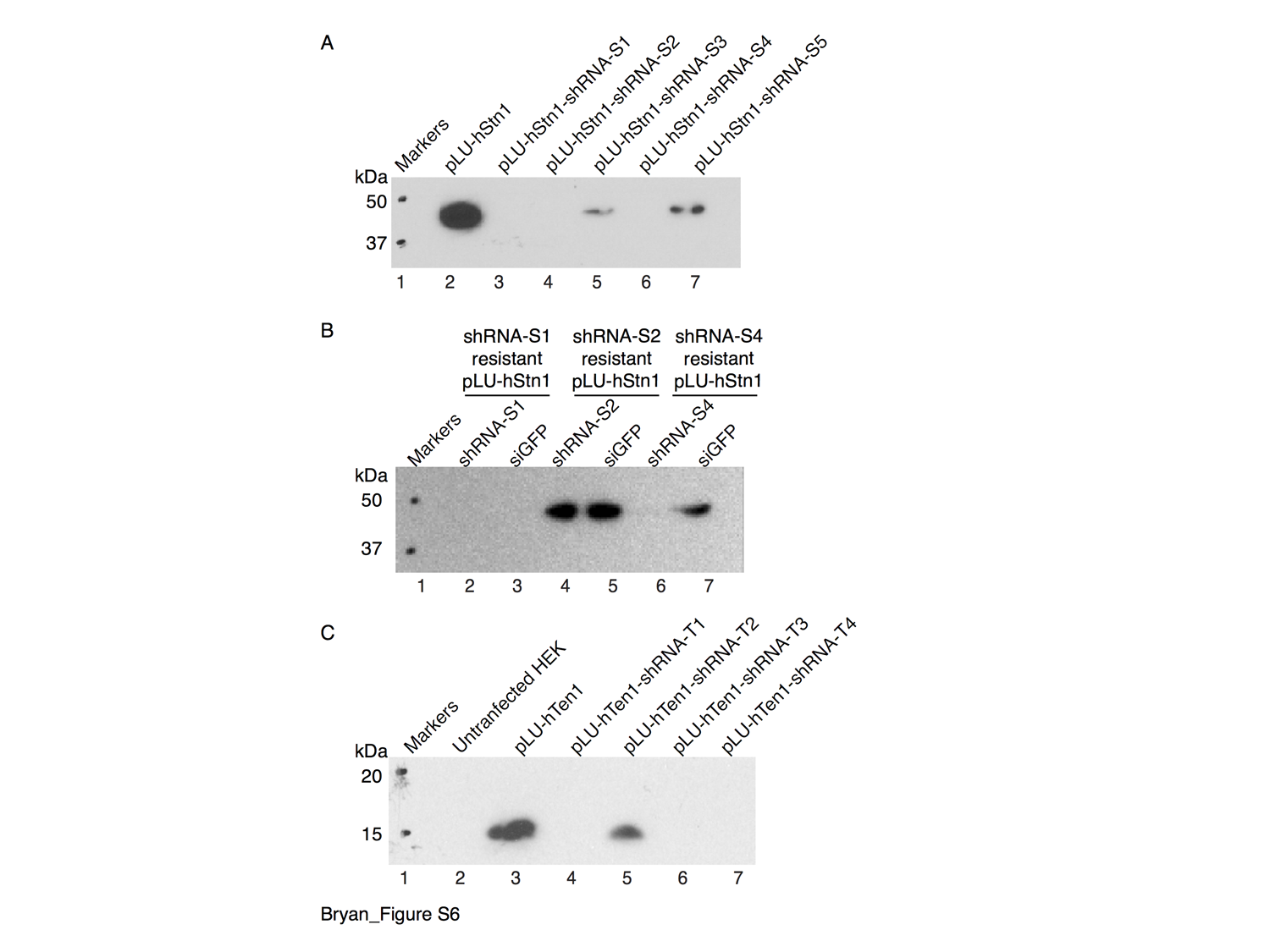

Supplement: Figure S6 — Western blots analysis of hStn1 and hTen1 expression in HEK 293T cellines. (A) Anti-flag tag Western blot showing the effect of five hStn1 shRNAs on ectopic hStn1 overexpression in the HEK 293T cells. Lanes: 1) Markers 2) hStn1 (WT) 3) shRNA-S1 4) shRNA-S2 5) shRNA-S3 6) shRNA-S4 and 7) shRNA-S5. (B) Anti-flag Western blot testing shRNA resistance of 3 different hStn1 genes carrying silent mutations designed to prevent binding of shRNA-S1, 2 and 4. Lane 1) Markers; Lanes 2–7, HEK 293T cells co-infected with 2) shRNA-S1 resistant hStn1 3) siGFP and shRNA-S1 resistant hStn1 4) shRNA-S2 resistant hStn1 5) siGFP and shRNA-S2 resistant hStn1 6) shRNA-S4 resistant hStn1 7) siGFP and shRNA-S4 resistant hStn1 (C) Anti-flag tag Western blot showing the effect of anti-hTen1 shRNAs on ectopic hTen1 overexpression in the HEK 293T cells. Lanes: 1) Markers 2) untransfected HEK 293T cells 3) hTen1-pLU 4) shRNA-T1 5) shRNA-T2 6) shRNA-T3 and 7) shRNA-T4. (TIF) [file pone.0066756.s006.tif]
